# Pan-Immune-Inflammation Value as a Novel Prognostic Biomarker for Advanced Pancreatic Cancer

**DOI:** 10.7759/cureus.71251

**Published:** 2024-10-11

**Authors:** Asim Armagan Aydin, Erkan Kayikcioglu, Ahmet Unlu, Mehmet Acun, Halil Goksel Guzel, Ridvan Yavuz, Halit Ozgul, Arif Hakan Onder, Banu Ozturk, Mustafa Yildiz

**Affiliations:** 1 Department of Clinical Oncology, Health Science University Antalya Education and Research Hospital, Antalya, TUR; 2 Department of Clinical Oncology, Istinye University School of Medicine, Istanbul, TUR; 3 Department of Surgical Oncology, Health Science University Antalya Education and Research Hospital, Antalya, TUR

**Keywords:** biomarker, gastrointestinal tract tumors, metastasis, pancreatic cancer, pan-immune-inflammation value, prognosis, survival

## Abstract

Aim: This study investigated the prognostic value of the pan-immune-inflammation value (PIV) in patients with advanced-stage pancreatic cancer (PC).

Materials and methods: The cohort comprised 71 patients, with a median age of 65 years (range: 37-83). The majority (69%) of patients received the FOLFIRINOX regimen as first-line therapy. Using ROC curve analysis, PIV demonstrated high diagnostic accuracy in predicting mortality, with an area under the curve (AUC) of 0.84 (95% CI: 0.72-0.97) and an optimal cut-off point of 276.5.

Results: Elevated PIV was significantly associated with mortality (p = 0.014), and patients with high PIV exhibited significantly poorer overall survival (OS) and progression-free survival (PFS) than those with low PIV (OS: 9.0 months vs. 26.0 months, p < 0.001; PFS: 7.0 months vs. 15.0 months, p < 0.001). Univariate and multivariate analyses identified PIV and the selected chemotherapy regimens as independent prognostic factors for OS and PFS.

Conclusion: High PIV values are associated with worse clinical outcomes, reinforcing its role as a reliable prognostic biomarker in advanced-stage PC. These findings underscore the importance of PIV in guiding therapeutic strategies and warrant further investigation in larger cohorts.

## Introduction

Pancreatic cancer (PC), with its increasing incidence and high mortality rates globally, is identified as the seventh leading cause of cancer-related deaths by 2024 [1]. This malignancy is notably aggressive, with an estimated global incidence of 10.1 cases per 100,000 individuals annually, a rate that is significantly higher in more developed countries [2,3]. The late-stage diagnosis of the disease and the limited surgical options available contribute adversely to survival rates. At diagnosis, approximately 50-60% of patients with PC present with metastatic disease, yielding a median overall survival (OS) of approximately six months [3]. Recent advancements, including the FOLFIRINOX regimen and the combination of nab-paclitaxel and gemcitabine, have only modestly increased median survival to approximately 12 months in advanced-stage patients [4,5]. Additionally, variations in treatment response and associated side effects remain substantial challenges in the management of this malignancy. Despite well-known predictive markers such as (BReast CAncer) BRCA gene mutation and microsatellite instability (MSI) [6], and prognostic markers such as CA 19-9, lactate dehydrogenase (LDH), neutrophil-to-lymphocyte ratio (NLR) [7], and Kirsten rat sarcoma virus (K-RAS) gene mutations [8], there remains a critical need for non-invasive biomarkers that can facilitate the development of personalized treatment strategies with the ability to predict clinical outcomes in PC.

Immune inflammation, which is the inflammatory response of the immune system, plays a crucial role in cancer prognosis [9]. Throughout cancer development and progression, the immune system not only provides a protective response against tumor cells but may also contribute to tumor growth through chronic inflammation [10]. This duality has led to various scientific insights into the effect of immune inflammation on cancer prognosis. The tumor microenvironment (TME) is a complex ecosystem characterized by interactions between tumor cells, immune cells, stromal cells, and inflammatory mediators [11]. Chronic inflammation can foster an immunosuppressive milieu within the TME, enabling tumor cells to evade the immune system and develop a more aggressive phenotype [12]. In addition, a robust inflammatory response can facilitate tumor immune evasion [12]. Key components of peripheral blood, including platelets, neutrophils, monocytes, and lymphocytes, each exhibit unique properties that significantly influence the immune system and play crucial roles in the inflammatory response [13]. Cytokines and chemokines produced by these cells, in conjunction with acute-phase proteins synthesized via various cellular mechanisms (such as C-reactive protein, fibrinogen, and albumin), are essential in orchestrating the body's inflammatory response. However, inflammatory cytokines released by tumor cells can activate immunosuppressive cells, diminishing the efficacy of the immune system against tumors [14]. This accelerates cancer progression and metastasis [10,14]. Moreover, elevated levels of immune inflammation markers are associated with poorer responses to therapies, such as chemotherapy and immunotherapy.

The pan-immune-inflammation value (PIV) represents a readily calculable biomarker derived from a formula that incorporates the proportions of various immune and inflammatory cells obtained through routine blood tests. Initially characterized by Fucà et al. [15], PIV has been linked to diminished OS in patients with metastatic colorectal cancer. Subsequent investigations have extensively explored the prognostic significance of PIV in breast [16], prostate [17], gastric [18], and esophageal cancers [19] and have consistently demonstrated the potential of PIV as a predictive biomarker for treatment efficacy and clinical outcomes. Furthermore, the results of a comprehensive meta-analysis, which included various cancer types treated with immune checkpoint inhibitors (ICIs), also support the notion that elevated PIV is linked to poorer survival outcomes [20]. Collectively, these findings indicate that PIV may serve as a promising tool for enhancing personalized treatment strategies and optimizing clinical outcomes in cancer management through more precise decision-making.

In the present study, we conducted a comprehensive analysis of the associations between PIV and clinicopathological characteristics of PC, as well as its influence on survival outcomes. Given that PIV has demonstrated prognostic potential across numerous studies focusing on cancer prognosis, elucidating its role in the clinical outcomes of advanced-stage PC may offer critical insights into risk stratification and optimization of therapeutic strategies. These findings have the potential to enhance both the management and prognosis of patients with this highly aggressive malignancy. To our knowledge, this is the first study to assess the prognostic and predictive implications of PIV in patients with advanced-stage PC.

## Materials and methods

Study design, patient selection, and data collection

**Figure 1:**
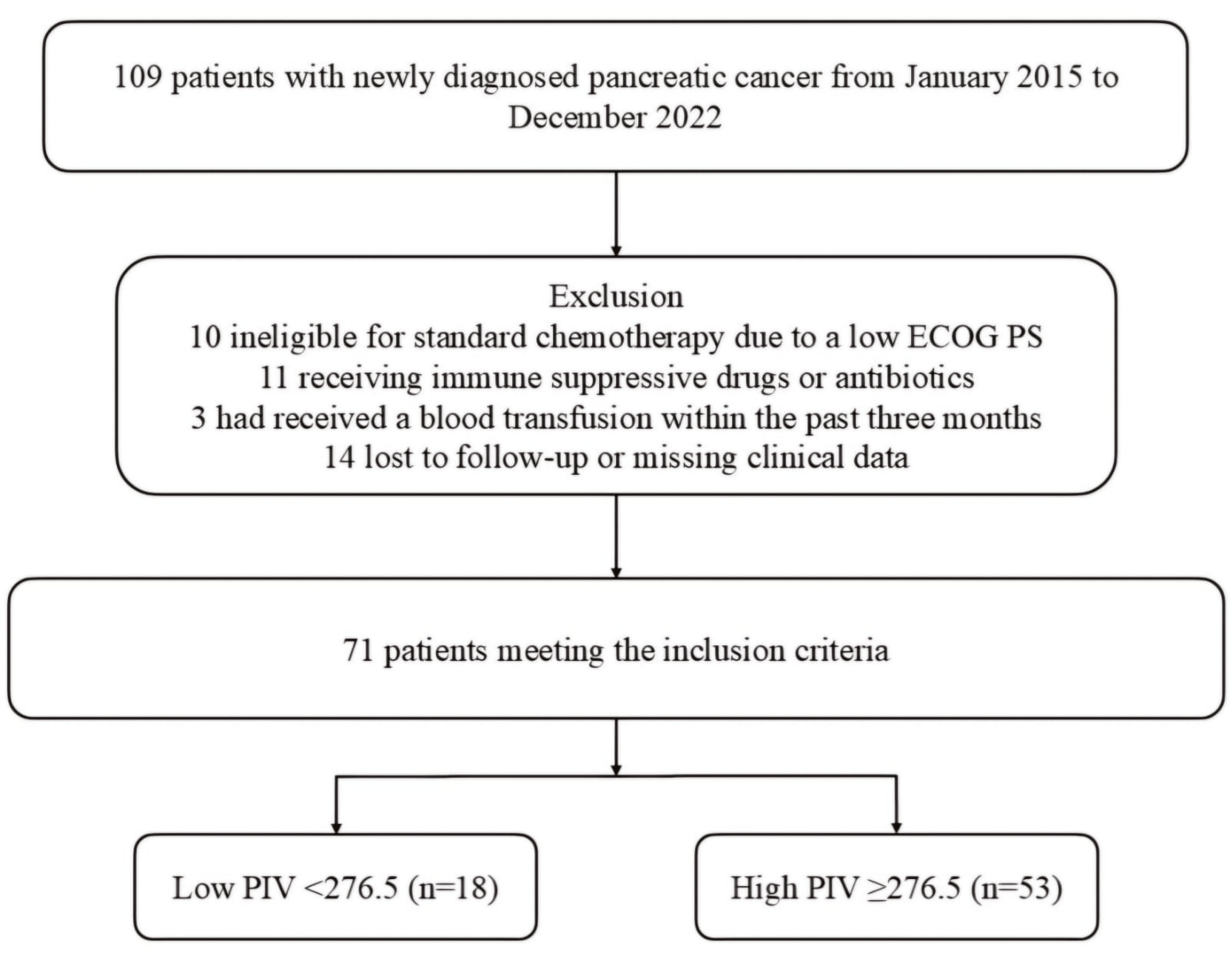
Flowchart of the study according to CONSORT diagram PIV: pan-immune-inflammation value; ECOG PS: Eastern Cooperative Oncology Group Performance Status; CONSORT: consolidated standards of reporting trials

Following ethical approval from the Institutional Ethics Committee (approval number: 2024-249), 109 patients treated at the Clinical Oncology Department of Health Sciences University Antalya Education and Research Hospital (HSUAERH) with a pathologically confirmed diagnosis of PC between January 2015 and December 2022 were retrieved from archival records. Ten patients who were ineligible for standard chemotherapy due to a low Eastern Cooperative Oncology Group performance status (ECOG PS) (>2), 11 patients with a history of prolonged immunosuppressive treatment due to chronic immune or inflammatory conditions or antibiotic use, 3 patients who had received a blood transfusion within the past three months, and 14 patients with incomplete medical laboratory or radiological data during the clinical follow-up were excluded from the study. In conclusion, 71 patients who adhered to the core design and met all the study criteria were included in the final analysis (Figure 1).

Following a thorough review of the clinical, laboratory, and radiological records of the patients, the following data were systematically collected: age, sex, ECOG PS, body mass index (BMI), comorbidities, smoking status, alcohol consumption, presence of obesity, presence of diabetes, tumor location, metastatic involvement (including liver, lung, and peritoneum), selected chemotherapy regimen, the occurrence of neutropenia during systemic chemotherapy, response to the conventional chemotherapy regimen, progression during follow-up, selected treatment options at the time of progression, and OS duration.

As illustrated in the schematic below, the PIV was computed utilizing the formula established by Fucà et al. [15]: \[PIV = \frac{N \times P \times M}{L}\]

where N represents neutrophils, P represents platelets, M represents monocytes, and L represents lymphocytes.

Ethical considerations were rigorously upheld throughout this study, which was conducted in accordance with the principles outlined in the Helsinki Declaration of 1964 as revised in 2013. The study protocol underwent a comprehensive review and received approval from the Institutional Review Board of Health Science University Antalya Education and Research Hospital (approval number: 2024-249). Due to the retrospective nature of this study, obtaining informed consent from patients was not requisite. However, to ensure the protection of patient confidentiality, all data were anonymized.

Treatment details and response assessment

Subsequent to the preliminary clinical evaluation, all patients were administered treatment in accordance with the standard protocol delineated by the National Comprehensive Cancer Network (NCCN). This protocol encompassed conventional chemotherapy regimens, specifically FOLFIRINOX (a composite treatment involving leucovorin, fluorouracil, irinotecan, and oxaliplatin) or gemcitabine-based therapies, which included combinations of gemcitabine with nab-paclitaxel or cisplatin. Comprehensive biochemical tests, including complete blood count, uric acid, LDH, albumin, and CRP levels, were performed concurrently within 7-10 days prior to the initiation of systemic chemotherapy. Following the completion of systemic chemotherapy, a response assessment was conducted using positron emission tomography with 2-deoxy-2-(fluorine-18) fluoro-D-glucose integrated with computed tomography (18F-FDG PET/CT). Clinical responses were assessed and categorized as complete response (CR), partial response (PR), stable disease (SD), or progressive disease (PD), according to the revised Response Evaluation Criteria in Solid Tumors (RECIST) guidelines (version 1.1). Progression-free survival (PFS) was defined as the time elapsed from the date of pathological diagnosis to the date of progression, death, or the last visit. OS was calculated as the time elapsed from the date of pathological diagnosis to the date of death from any cause or the last visit.

Statistical analysis

Statistical analyses were performed using IBM SPSS Statistics for Windows, Version 27 (Released 2020; IBM Corp., Armonk, New York, United States). The normal distribution suitability of continuous data was evaluated using the Kolmogorov-Smirnov and Shapiro-Wilk tests. Numerical variables conforming to a normal distribution are expressed as mean ± standard deviation, whereas those deviating from normality are presented as median (min-max). The predictive accuracy of PIV for mortality was evaluated using receiver operating characteristic (ROC) curve analysis. The optimal cut-off value for the PIV ratio was determined using the Youden Index method within the ROC curve analysis. Continuous data were compared using the independent samples t-test or Mann-Whitney U test. Categorical data were compared using Pearson’s chi-squared test. Fisher’s exact test was used when expected value problems occurred. PFS and OS were estimated using the Kaplan-Meier method and compared using the log-rank test. Variables significantly associated with survival in univariate analysis were further analyzed using multivariate Cox regression models. Statistical significance was defined as p < 0.05 for all analyses.

## Results

The median age of the cohort was 65 years (range: 37-83 years). Thirty-six patients (50.7%) were aged >65 years, and 45 patients (63.4%) were male. A history of smoking was documented in 35 patients (49.3%), whereas 14 patients (19.7%) reported a history of alcohol consumption. A total of 31% of the patient cohort presented with diabetes, and 7% were classified as obese (BMI >30 kg/m^2^). The ECOG PS scores were 0-1 in 50 patients and 2 in 21 patients. All the patients exhibited tumors with adenocarcinoma morphology. The pancreatic head was the most common tumor location, identified in 54.9% of cases. The liver (70.4%), peritoneum (22.5%), and lungs (15.5%) were the most frequently affected sites of metastasis. In terms of first-line therapy, most patients (69%) underwent treatment with the FOLFIRINOX regimen. Responses to first-line chemotherapy were assessed according to the RECIST Criteria in Solid Tumors. PD was observed in 15 patients (21.1%), SD in 27 (38.0%), PR in 21 (29.6%), and CR in eight (11.3%). Table 1 presents a comprehensive summary of the demographic and clinical characteristics of patients with advanced PC, categorized by their PIV levels.

**Table 1:** Comparison of sociodemographic and clinicopathological characteristics of patients with advanced pancreatic cancer classified according to the PIV (all patients, n = 71) ECOG PS: Eastern Cooperative Oncology Group Performance Status; PIV: pan-immune-inflammation value ^*^statistically significant (p < 0.05)

Variables			PIV		p-value^*^
			Low (<276.5)	High (≥276.5)	
Age (year), n (%)	<65	35 (49.3)	11 (61.1)	24 (45.3)	0.188
	≥65	36 (50.7)	7 (38.9)	29 (54.7)	
Sex, n (%)	Female	26 (36.6)	7 (38.9)	19 (35.8)	0.515
	Male	45 (63.4)	11 (61.1)	34 (64.2)	
ECOG PS, n (%)	0-1	50 (70.4)	13 (72.2)	37 (69.8)	0.55
	2	21 (29.6)	5 (27.8)	16 (30.2)	
Smoking status, n (%)	None	36 (50.7)	9 (50.0)	27 (50.9)	0.58
	Present	35 (49.3)	9 (50.0)	26 (49.1)	
Diabetes mellitus, n (%)	None	49 (69.0)	13 (72.2)	36 (67.9)	0.489
	Present	22 (31.0)	5 (27.8)	17 (32.1)	
Alcohol consumption, n (%)	None	57 (80.3)	15 (83.3)	42 (79.2)	0.501
	Present	14 (19.7)	3 (16.7)	11 (20.8)	
Obesity, n (%)	None	66 (93.0)	18 (100.0)	48 (90.6)	0.22
	Present	5 (7.0)	0 (0.0)	5 (9.4)	
Comorbidity, n (%)	None	35 (49.3)	11 (61.1)	24 (45.3)	0.188
	Present	36 (50.7)	7 (38.9)	29 (54.7)	
Tumor location, n (%)	Head	39 (54.9)	11 (61.1)	28 (52.8)	0.699
	Body	16 (22.5)	3 (16.7)	13 (24.5)	
	Tail	16 (22.5)	4 (22.2)	12 (22.6)	
Liver metastasis, n (%)	None	21 (29.6)	6 (33.3)	15 (28.3)	0.45
	Present	50 (70.4)	12 (66.7)	38 (71.7)	
Lung metastasis, n (%)	None	60 (84.5)	16 (88.9)	44 (83.0)	0.432
	Present	11 (15.5)	2 (11.1)	9 (17.0)	
Peritoneal involvement, n (%)	None	55 (77.5)	15 (83.3)	40 (75.5)	0.369
	Present	16 (22.5)	3 (16.7)	13 (24.5)	
Chemotherapy regimen, n (%)	FOLFIRINOX	49 (69.0)	15 (83.3)	34 (64.2)	0.139
	Cisplatin plus Gemcitabine	13 (18.3)	2 (11.1)	11 (20.8)	
	Nab-paclitaxel plus Gemcitabine	9 (12.7)	1 (5.6)	8 (15.1)	
Progression, n (%)	None	2 (2.8)	1 (5.6)	1 (1.9)	0.445
	Present	69 (97.2)	17 (94.4)	52 (98.1)	
Death, n (%)	None	3 (4.2)	3 (16.7)	0 (0.0)	0.014
	Present	68 (95.8)	15 (83.3)	53 (100.0)	

Cut-off values of the laboratory parameters

**Figure 2:**
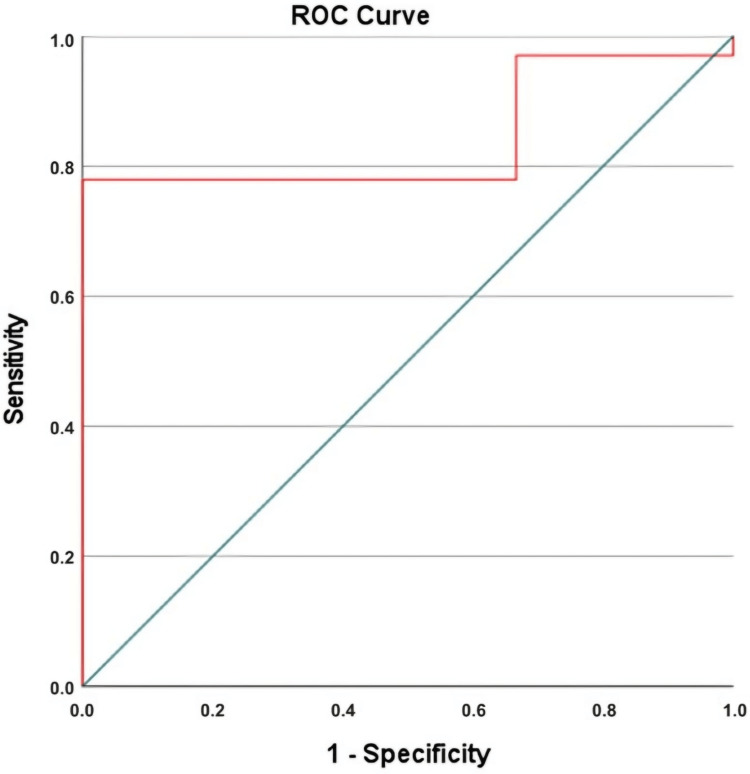
An illustration of the predictive capability of the PIV for mortality in advanced-stage pancreatic cancer using ROC curve analysis ROC: receiver operating characteristic; PIV: pan-immune-inflammation value

The diagnostic accuracy of the laboratory parameters in predicting mortality prior to chemotherapy in patients with advanced-stage PC was assessed using ROC curve analysis (Figure 2). The highest area under the ROC curve (AUC) for the PIV was established at 0.84 (95% CI: 0.72-0.97) (Table 2). The optimal cut-off point for PIV, determined by the maximum Youden Index, was 276.5. Notably, all patients with elevated PIV values died, and a statistically significant association between PIV and mortality was observed (p = 0.014). However, no significant correlations were observed between PIV and other clinical variables (p > 0.05, Table 1).

**Table 2:** AUC value for PIV compared using ROC curve analysis AUC: area under the curve; Std: standard deviation; CI: confidence interval; ROC: receiver operating characteristic; PIV: pan-immune-inflammation value

AUC	Std. error	%95 CI (lower-upper)		Specificity	Sensitivity	p-value
0.843	0.065	0.716	0.970	77.9%	66.7%	0.045

Survival analysis

**Figure 3:**
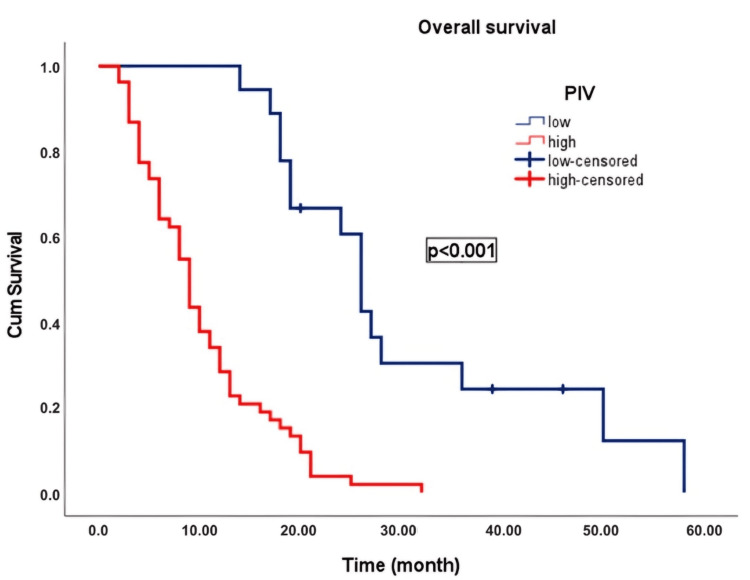
Kaplan-Meier curve illustrating the overall survival of patients with advanced-stage pancreatic cancer classified according to the PIV PIV: pan-immune-inflammation value

**Figure 4:**
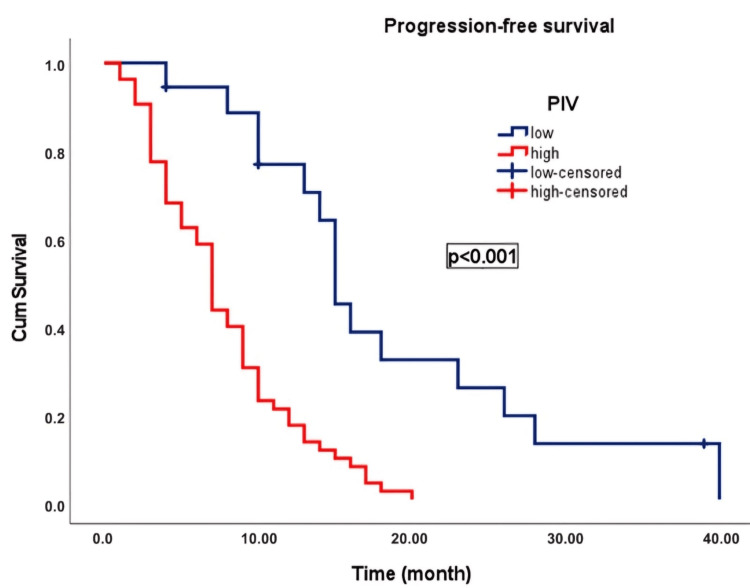
Kaplan-Meier curve illustrating the progression-free survival of patients with advanced-stage pancreatic cancer classified according to the PIV PIV: pan-immune-inflammation value

In an average follow-up duration of 17.5 months (95% CI: 4.1-56.0), progression occurred in 69 patients (97.2%), and 68 patients (95.8%) died. In patients with advanced-stage PC, the median OS was 12.0 (2-58 months). OS was 26.0 ± 1.3 months (23.4-28.6) in patients with low PIV and 9.0 ± 0.7 months (7.6-10.4) in patients with high PIV. Patients with low PIV exhibited significantly longer OS than those with high PIV (p < 0.001) (Figure 3). The median PFS was 9.0 (1-40 months). PFS was 15.0 ± 0.7 (13.7-16.3) months in patients with low PIV and 7.0 ± 0.5 (6.1-7.9) months in patients with high PIV. Patients with low PIV scores exhibited significantly longer PFS than those with high PIV scores (p < 0.001) (Figure 4). The Kaplan-Meier survival curves for OS and PFS stratified by the low and high PIV groups are shown in Figures 3, 4, respectively.

The clinical and laboratory parameters affecting the OS of patients with advanced-stage PC were investigated using a univariate Cox proportional hazards model (Table 3). In the univariate analysis, the selected chemotherapy regimen (p = 0.008), and PIV (p < 0.001) were found to be significantly associated with OS.

**Table 3:** Univariate Cox regression analyses for OS in patients with advanced-stage pancreatic cancer ECOG PS: Eastern Cooperative Oncology Group Performance Status; PIV: pan-immune-inflammation value; OS: overall survival; HR: hazard ratio ^*^The administration of FOLFIRINOX as first-line treatment provides favorable data regarding OS

	Overall survival			
	Univariate			
	HR (95% CI for HR)			p-value
Age	1.616	0.982	2.660	0.059
Sex	0.953	0.580	1.567	0.851
ECOG PS	1.209	0.708	2.063	0.487
Smoking	0.864	0.533	1.399	0.551
Alcohol use	0.993	0.528	1.868	0.983
Diabetes	1.314	0.779	2.216	0.305
Obesity	2.112	0.820	5.437	0.121
Comorbidity	1.304	0.805	2.112	0.281
Tumor location	1,035	0.776	1.382	0.813
Liver metastasis	1.172	0.698	1.968	0.548
Lung metastasis	1.238	0.644	2.381	0.522
Peritoneal involvement	0.860	0.476	1.555	0.617
Selected chemo regimen^*^	1.634	1.136	2.351	0.008
PIV	5.753	2.907	11.383	<0.001

In the univariate Cox proportional hazards model, age, selected chemotherapy regimen, and PIV were significantly associated with PFS (Table 4).

**Table 4:** Univariate Cox regression analyses for PFS in patients with advanced-stage pancreatic cancer ECOG PS: Eastern Cooperative Oncology Group Performance Status; PIV: pan-immune-inflammation value; HR: hazard ratio ^*^The administration of FOLFIRINOX as first-line treatment provides favorable data regarding PFS

	Progression-free survival			
	Univariate			
	HR (95% CI for HR)			p-value
Age	1.958	1.194	3.210	0.008
Sex	0.941	0.572	1.549	0.812
ECOG PS	1.432	0.843	2.432	0.184
Smoking	1.003	0,620	1.624	0.989
Alcohol use	1.070	0.582	1.969	0.827
Diabetes	1.341	0.796	2.260	0.270
Obesity	1.803	0.706	4.606	0.218
Comorbidity	1.379	0.849	2.241	0.195
Tumor location	0.987	0.741	1.316	0.931
Liver metastasis	1.651	0.967	2.818	0.066
Lung metastasis	1.045	0.545	2.004	0.894
Peritoneal involvement	0.933	0.521	1.673	0.817
Selected chemo regimen^*^	1.740	1.194	2.534	0.004
PIV	3.982	2.033	7.798	<0.001

In multivariate analysis, the selected chemotherapy regimen and PIV were significantly associated with OS (p = 0.018 and p < 0.001, respectively). In addition, significant associations with PFS were found for age, selected chemotherapy regimen, and PIV (Table 5).

**Table 5:** Multivariate Cox regression analyses for OS and PFS in patients with advanced-stage pancreatic cancer PIV: pan-immune-inflammation value; OS: overall survival; HR: hazard ratio; PFS: progression-free survival ^*^The administration of FOLFIRINOX as a first-line treatment provides favorable data regarding both OS and PFS

	Multivariate								
	Overall survival					Progression-free survival			
	HR (95% CI for HR)			p-value		HR (95% CI for HR)			p-value
Age	-	-	-	-	Age	1.676	1.010	2.779	0.046
Selected chemo regimen^*^	1.586	1.083	2.323	0.018	Selected chemo regimen^*^	1.550	1.059	2.269	0.024
PIV	5.689	2.864	11.301	<0.001	PIV	3619	1821	7194	<0.001

Both univariate and multivariate analyses indicated that high PIV and the chosen chemotherapy regimen, excluding FOLFIRINOX, were adverse prognostic factors associated with reduced OS and PFS. An elevated PIV value independently serves as a risk factor for both OS and PFS in patients with advanced-stage PC. Furthermore, it was a robust predictor of adverse clinical outcomes.

## Discussion

PIV plays a pivotal role in the understanding of the biological mechanisms underlying PC progression and metastasis. Elevated PIV levels are indicative of a heightened systemic inflammatory response, which is known to contribute to the microenvironment's dynamics [9]. In PC, this inflammatory milieu promotes tumor growth and dissemination by promoting angiogenesis, suppressing effective immune responses, and facilitating epithelial-to-mesenchymal transition (EMT), which is a key process associated with metastatic potential [10]. Moreover, inflammatory cells, such as macrophages and neutrophils, within the TME secrete cytokines and growth factors that further enhance tumor aggressiveness and facilitate local invasion [14]. Thus, the interplay between PIV and inflammatory processes not only influences cancer progression but also serves as a crucial factor in the metastatic spread of PC, highlighting the need for targeted therapeutic strategies to address this inflammatory axis.

The findings of this study support the notion that high PIV values in patients with advanced-stage PC are strongly associated with poor OS and PFS. Univariate and multivariate analyses indicated that PIV had a strong predictive effect on clinical outcomes. Based on the data derived from the regression analysis, the association between PIV and key factors that directly affect survival, such as advanced age and the use of the FOLFIRINOX regimen as first-line treatment, further underscores its prognostic capability. This effect may stem from PIV design, which integrates multiple markers, allowing it to more accurately reflect the connection between immune inflammation and cancer dynamics compared to indices based on binary formulations, such as the NLR, monocyte-to-lymphocyte ratio (MLR), and platelet-to-lymphocyte ratio (PLR). This, in turn, strengthens the predictive potential of PIV. The straightforward and accessible nature of calculating the PIV using routine blood tests, combined with its non-invasive and cost-effective attributes, positions it as a compelling candidate for incorporation into clinical practice. Its application could offer clinicians a valuable tool for risk stratification and treatment selection in patients with PC. In conclusion, PIV has emerged as a significant biomarker that reflects the adverse impact of systemic inflammation on prognosis in advanced-stage PC. Its potential to inform future research and clinical decision-making in this context has been highlighted, warranting further investigation. 

The prognostic significance of PIV in cancer has been extensively investigated for various malignancies. PIV was initially introduced by Fucà et al., who demonstrated that elevated pre-treatment PIV levels were predictive of poor prognosis in colorectal cancer [15]. Subsequent studies have consistently shown that high pre-treatment PIV values are associated with diminished survival outcomes in advanced HER2-positive breast cancer, metastatic castration-resistant prostate cancer, advanced gastric cancer treated with ICIs, and resectable esophageal cancer [16-19]. Moreover, a meta-analysis by Kuang et al., which included 982 cancer patients treated with ICIs, further reinforced the predictive value of elevated PIV in terms of worsened PFS and OS [20]. A broader meta-analysis by Guven et al., encompassing 15 studies and 4,942 cancer patients, similarly underscored the role of PIV as a robust prognostic biomarker in oncology [21]. Additionally, recent findings by Topkan et al. in a cohort of patients with locally advanced PC demonstrated that high PIV values before chemoradiotherapy were significantly correlated with adverse prognostic outcomes [22]. The results of our study are in concordance with these existing findings, further substantiating the relevance of PIV in cancer prognosis. To the best of our knowledge, this study is the first to evaluate the prognostic significance of PIV, specifically in patients with advanced-stage PC. These findings may serve as a basis for future prospective studies in this area, contributing to a deeper understanding of the role of PIV in cancer prognoses.

The present study is subject to several limitations. First, the retrospective design of this study, along with its relatively small cohort size and single-center focus, may impact the equitable distribution of cases, the application of more advanced statistical analyses, and the generalizability of the findings. The index utilized in this research, derived from a multivariate formula, incorporates markers that may influence each other indirectly. Furthermore, some of these markers have the potential to activate intrinsic chemokines or cytokines, subsequently influencing immune responses and the clinical trajectory of cancer through diverse mechanisms. Additionally, the absence of a universally established standard cutoff value for the PIV represents a further limitation. Certain confounding factors, such as subclinical infections present at the time of parameter measurement, individual variability in immune system alterations, transient fluctuations in marker levels, and the lack of an internal validation cohort, may also have been overlooked. Moreover, potential bias related to differences in advanced-line treatment options between PIV groups should be considered. Future research involving larger, multicenter cohorts and incorporating internal validation groups is recommended to provide more robust and reliable insights into the prognostic and predictive value of PIV.

## Conclusions

In summary, this study highlights the significant prognostic value of the PIV in patients with advanced-stage PC. Our findings indicate that elevated PIV levels are associated with poorer OS and PFS, emphasizing the influence of systemic inflammation on cancer progression. The PIV’s capacity to integrate multiple biomarkers enhances its predictive power, rendering it a promising tool for clinical application. Nonetheless, certain limitations, such as the retrospective design, small sample size, and the absence of standardized cutoff values, underscore the necessity for further research into the role of PIV in cancer prognosis. Future investigations involving larger cohorts and internal validation are essential to deepen our understanding of its utility and to inform therapeutic strategies aimed at improving patient outcomes.
